# The hematopoietic saga of clonality in sickle cell disease

**DOI:** 10.1172/JCI158251

**Published:** 2022-03-01

**Authors:** Aaron J. Stonestrom, Ross L. Levine

**Affiliations:** 1 Human Oncology and Pathogenesis Program,; 2 Department of Medicine, Leukemia Service,; 3 Center for Epigenetics Research,; 4 Center for Hematologic Malignancies, and; 5 Molecular Cancer Medicine Service, Memorial Sloan-Kettering Cancer Center, New York, New York, USA.

## Abstract

Sickle cell disease (SCD) is associated with an increased risk of vascular-occlusive events and of leukemia. Clonal hematopoiesis (CH) may increase both risks. In turn, physiologic abnormalities in SCD may modify the incidence and/or distribution of genetic alterations in CH. In a recent issue of the *JCI*, Liggett et al. found no difference in CH rate between individuals with versus without SCD. Here we contextualize this report and discuss the complex interplay between CH and SCD with particular attention to consequences for emerging gene therapies. We further consider the limitations in our current understanding of these topics that must be addressed in order to optimize therapeutic strategies for SCD.

## Sickle cell disease presents specific challenges for hematopoiesis

**Figure 1:**
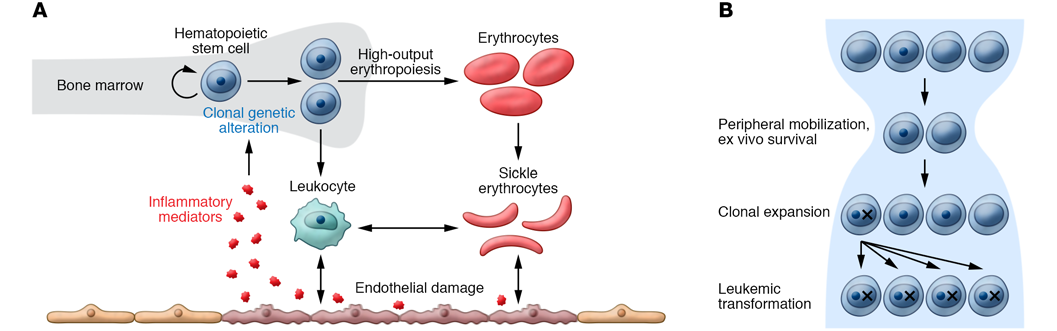
Clonal dynamics in sickle cell disease and its therapy. (**A**) High-output erythropoiesis and the inflammatory state of sickle cell disease may create an environment that favors hematopoietic clones with specific mutations. Mutant hematopoietic clones may also have increased potential to trigger sickling and inflammation. (**B**) Hematopoietic stem cell harvest and expansion may cause a bottleneck in the hematopoietic progenitor population followed by an expansion phase, both of which may favor mutant cells. This expanded proportion of mutant stem cells may place patients at high risk of leukemic transformation.

Sickle cell disease (SCD) is a monogenic blood disorder that affects millions of people worldwide. SCD is commonly caused by homozygosity for the *HBB* Glu6Val (rs334) (βS) genotype. This genotype causes production of hemoglobin that is predisposed to aggregate in the setting of hypoxia and deform erythrocytes into a sickle-like morphology. Salient clinical consequences include cell-intrinsic hemolytic anemia, high risk of vaso-occlusive events, including cerebrovascular accident, acute chest syndrome, and pain crises, as well as chronic systemic sequelae including pulmonary hypertension, nephropathy, and retinopathy (1). The short lifespan of erythrocytes in SCD creates a sustained need for increased red blood cell production and presumably a need for increased hematopoietic stem and progenitor cell (HSPC) proliferation. Both hemolysis and vaso-occlusive events precipitate inflammation, oxidative stress, and vascular-endothelial dysfunction (2). Although the risk of hematologic malignancy in SCD is low relative to other complications, it is substantially increased when compared with the general population (3, 4). Malignancy is likely a secondary consequence of SCD physiology because *HBB* expression is limited to differentiated erythroid elements that are unable to undergo oncologic transformation. High HSPC turnover and the action of inflammatory mediators have been postulated as pathogenetic factors (Figure 1).

Anecdotal reports have suggested that therapy with hydroxyurea (HU) may also increase the risk of leukemia in SCD. For nearly 30 years HU was the only FDA-approved therapy for SCD, and it remains a therapeutic mainstay despite the approval of new agents. HU increases expression of the protective fetal β-globin gene *HBF* and reduces complications of SCD. The therapeutic effect of HU is accomplished by inhibiting the enzyme ribonucleotide reductase, which is required for efficient DNA synthesis. Unfortunately, HU is broadly toxic to rapidly dividing cells, akin to DNA-damaging chemotherapy. While chemotherapy favors expansion of clonal hematopoiesis (CH) clones with mutations in DNA-damage repair genes, including *TP53* (5, 6), it is not known whether HU treatment has the same effect. Reassuringly, several large studies have found no increased risk of leukemia in SCD patients taking HU (7, 8).

## Myeloid leukemogenesis has complicated potentially curative cellular therapies

The only well-established curative therapy for SCD is allogenic hematopoietic stem cell transplantation (HSCT) (9). Patient-specific challenges including donor identification, transplant-related complications, and graft-versus-host disease are common to the allogenic HSCT field. Less expectedly, cases of acute myeloid leukemia (AML) developing from host cells following allogenic HSCT in patients with SCD have been reported (10, 11). AML in these patients had features strongly correlated with *TP53* mutation, including complex karyotype and large deletions involving chromosome 7. These genetic alterations are commonly associated with therapy-related myeloid neoplasms and confer a dismal prognosis. Intriguingly, in 2 patients, *TP53*-mutant CH was retrospectively detected in banked samples prior to development of leukemia, albeit at low variant allele frequency (VAF) (0.34% and 0.06%). While *TP53*-mutant CH can also be found in healthy individuals in the general population, it is rare in younger individuals (as these transplant recipients were), and when identified it is associated with a high risk of progression to AML (5, 12, 13).

SCD has been at the forefront of gene therapy for reasons including the relative ease of making genetic alterations in the hematopoietic system, the single genetic alteration responsible, the insufficiency of current therapies to prevent serious complications, and high disease prevalence. Multiple clinical trials have utilized a general strategy of collecting HSPCs from patient peripheral blood, performing genetic manipulation ex vivo, treating patients with conditioning chemotherapy to facilitate engraftment, and reinfusing modified cells. The longest reported followup is with the strategy of expressing anti-sickling βA-T87Q from a lentiviral vector (HGB-206 trial). Notably, leukemia has developed in some early clinical trial participants (14, 15). The two reported cases of AML arising in HGB-206 were characterized by monosomy 7 and mutations in *RUNX1* and *PTPN11*. Lentiviral integration was detectable in leukemic blasts in only one of the two cases, and in this case extensive molecular investigation failed to find evidence that lentiviral integration contributed to leukemogenesis. This finding raises the specter that other gene therapy approaches (including sophisticated CRISPR-based gene editing to reactivate expression of *HBF* while switching off pathogenic *HBB*) may be at risk of similar complications.

Reports of AML in SCD patients resonate with a recent demonstration that autologous and allogeneic HSCT creates a microevolutionary bottleneck, selecting for HSPC clones with enhanced fitness due to mutation (ref. 16 and Figure 1). There is a possibility that the medley of high-risk CH and HSCT incites mutant clones to expand and subsequently transform. A strategy of testing for high-risk CH prior to therapeutic intervention and deferring gene therapy if found might abrogate the risk of leukemic transformation. Error-corrected sequencing can detect very low levels of myeloid leukemia (17) and could be considered in this setting. As such, it will be critical to fully understand the risk of CH in patients with SCD, and to identify whether there are patient subsets at higher risk, such as those with severe disease, chronically high inflammatory markers, or those who have received particular therapies.

## Incidence of CH in SCD

Although CH is associated with both an increased risk of AML and an increased risk of cardiovascular events, for most people the consequences of CH are minimal. However, there is high potential for these CH-associated risks to synergize with the underlying risks of SCD. Two recent studies have addressed CH in SCD.

One study (18) analyzed CH in whole-exome sequencing data from 1,459 patients with SCD using an adjacent set of 6,848 African Americans as a comparator. In this study, subjects were considered to have CH if they had an alteration in any of 46 handpicked genes with VAF greater than or equal to 2% and less than 40%. Fifteen CH mutations in 15 individuals with SCD were identified. Provocatively, the authors found a higher risk of CH with SCD, with an odds ratio point estimate of 13.5 using a model that accounted for age and other confounders. Notably, average sequencing depth was higher in the SCD patients than in controls; however, a sensitivity analysis suggested that bias in sequencing depth did not substantially change the result.

Liggett et al. (19) used whole-genome sequencing (WGS) data from the NHLBI TOPMed consortium (20) to characterize CH in 3,090 individuals affected by SCD and 71,100 unaffected individuals. CH calls were made in a set of leukemia-driver mutations using an established software pipeline (GATK Mutect2). The authors estimated they were able to detect half of variants with VAF 5% to 10% and nearly all variants with VAF greater than or equal to 10%. In contrast to the earlier findings referenced above, the authors did not detect an increased risk of CH in SCD, although there was a trend in this direction. Although the rate of CH reported by Liggett et al. (19) in SCD was slightly lower, it appears that difference in risk in each control group drove most of the difference in the results of the two studies. Comparison of the distribution of specific genes and VAFs between the two studies could further inform comparison.

Liggett et al. (19) also evaluated the relative risk of CH in patients with SCD treated with HU and found that HU-treated SCD patients did not have an increased risk of CH relative to those not treated with HU. Despite unavoidable confounders inherent to this comparison it is very reassuring that HU was not associated with an increased risk of CH.

## Conclusions

The frequency and clinical implications of CH may differ in individuals with and without SCD due to differences in hematopoietic physiology. Recent reports describing the risk of CH in SCD have reached different conclusions about its relative incidence. Liggett et al.’s (19) work supports the notion that the prevalence of CH in individuals with SCD may be more similar to that of the general population than was previously thought. If true, the increased risk of AML observed in SCD may come from low-VAF CH clones that were undetectable by the methods used. Large, carefully controlled studies of CH using highly sensitive assays are needed to understand how CH impacts SCD outcomes, to identify high-risk subgroups, and to determine whether screening for CH in SCD patients can reduce the risks associated with stem cell and gene therapies.

## Version 1 03/01/2022

Electronic publication

## Version 2 02/17/2022

In-Press Preview
